# Special Issue: Exploring Therapeutic Targets in the Evolving Landscape of Cancer Immunotherapy

**DOI:** 10.3390/ijms27010243

**Published:** 2025-12-25

**Authors:** Domenico Galati

**Affiliations:** Hematology-Oncology and Stem-Cell Transplantation Unit, Department of Onco-Hematology and Innovative Diagnostics, Istituto Nazionale Tumori–IRCCS-Fondazione “G. Pascale” IRCCS, 80131 Napoli, Italy; d.galati@istitutotumori.na.it

Cancer remains one of the primary causes of death worldwide, resulting in almost ten million fatalities per year [1,2,3,4,5]. While surgery, chemotherapy, radiotherapy, and hormone therapy continue to represent foundational pillars of care, their impact is limited by intrinsic and acquired resistance, systemic toxicity, and reduced feasibility in patients with advanced or frail conditions [2,3]. These limitations have long driven the development of complementary strategies, including immunologically based approaches that harness cytotoxic immune effector mechanisms and durable immunological memory to achieve sustained tumor control. While the interplay between immunity and cancer was discovered more than a century ago, only in recent decades have these concepts yielded broadly effective therapies such as immune checkpoint inhibitors and adoptive cellular therapies [1].

The underlying rationale is compelling. The immune system can discriminate malignant from healthy tissue; however, tumors develop multiple, layered mechanisms of immune escape, downregulating antigen presentation, secreting immunosuppressive cytokines such as interleukin-10 (IL-10) and transforming growth factor-β (TGF-β), recruiting regulatory T cells (Tregs) and myeloid-derived suppressor cells (MDSCs), and remodeling stromal elements to establish a permissive niche that dampens effective immune surveillance [6,7,8,9,10]. Overcoming tumor immune evasion has therefore become a central goal of modern oncology.

A further obstacle to effective antitumor immunity arises from the metabolic reprogramming of the tumor microenvironment (TME). Hypoxia, acidosis, and lactate accumulation compromise T-cell fitness and reinforce local immunosuppression, configuring a metabolic “lock” that resists immune attack [11]. In parallel, cancer-associated fibroblasts (CAFs) promote immune exclusion by secreting chemokines such as CXCL12 and remodeling the extracellular matrix, physically and functionally limiting the trafficking of effector lymphocytes into the tumor core [12].

Against this background, successful immunotherapy has been realized and is now widely regarded as the “fifth pillar” of cancer treatment, alongside surgery, radiotherapy, chemotherapy, and targeted agents [1,2,3,4,5]. A decade of clinical experience has revealed the breadth of available approaches, ranging from monoclonal antibodies and small-molecule immunomodulators to adoptive cell therapies, oncolytic viruses, and cancer vaccines, whilst also highlighting persistent challenges such as resistance and immune-related toxicity [13].

Immune checkpoint inhibitors have transformed the therapeutic landscape of melanoma, non-small-cell lung cancer (NSCLC), and several other malignancies. In the pivotal phase 3 trial of ipilimumab in previously treated metastatic melanoma, the best overall response rate was 10.9%, with disease control maintained for ≥2 years in approximately 60% of responders [6]. Early-phase anti-PD-1 studies in advanced melanoma, NSCLC, and renal cell carcinoma (RCC) reported objective response rates of approximately 20–25% [7], whereas PD-1 blockade achieved an ORR of 53% and a complete response rate of 21% in mismatch repair-deficient (MMRd) solid tumors [8]. Thus, objective responses remain confined to a minority of treated patients—often less than one-third in unselected advanced-disease cohorts—and many responders eventually relapse; nevertheless, these data reveal durable multi-year survival in heavily pretreated populations and suggest that the true potential of immune checkpoint-based therapy may be greater when it is used in first-line and earlier-stage settings [6,7,8,9]. Mechanistically, adaptive resistance has been linked to interferon-driven transcriptional programs that induce alternative inhibitory checkpoints and reduce antigen presentation, ultimately compromising the durability of response [14]. At the same time, emerging evidence suggests that the quality of neoantigens, encompassing clonality, intrinsic immunogenicity, and T-cell recognition potential, may be at least as pivotal as overall tumor mutational burden in forecasting benefit from checkpoint blockade [15].

Chimeric antigen receptor T-cell (CAR-T) therapy constitutes a second major breakthrough and differs conceptually from immunotherapies that primarily amplify intrinsic antitumor recognition. By engineering autologous T cells to recognize selected surface antigens, CAR-T therapies have achieved high remission rates in relapsed or refractory B-cell malignancies, with overall remission rates of around 80% in pediatric and young adult B-cell acute lymphoblastic leukemia and overall response rates of about 50% in adult relapsed or refractory diffuse large B-cell lymphoma [16,17]. However, translating these successes to solid tumors remains challenging. Antigen heterogeneity, poor trafficking across abnormal vasculature, and the risk of severe toxicities, including cytokine release syndrome and neurotoxicity, limit these therapies’ efficacy and adoption [18,19,20]. Because CAR specificities do not undergo physiological selection by central or peripheral tolerance mechanisms, these toxicities can be biologically novel and are closely linked to the same properties that underlie CAR-T potency. To address these issues, next-generation CAR designs are highly reliant on multi-omic antigen selection and context-dependent expression profiling, with the aim of reducing off-tumor toxicity and limiting immune escape [21]. In parallel, CAR-engineered natural killer (NK) cells are being developed as off-the-shelf cytotoxic platforms, offering potentially lower toxicity and complementary mechanisms of target recognition that broaden the horizon of cellular immunotherapy [22].

Beyond CAR-T, adoptive cell transfer (ACT) strategies and cancer vaccines benefit from more refined antigen discovery and improved vector technologies [6,7,8,9,10]. Notably, mRNA-based vaccines encoding patient-specific neoantigen repertoires have demonstrated encouraging immunogenicity in early trials and may prime or boost T-cell responses in immunologically “cold” tumors [23]. Overall, these developments point towards a more rational and multimodal use of immunotherapy, in which different approaches are combined and sequenced to achieve more durable tumor control.

Despite progress, several constraints continue to shape real-world practice. Primary and secondary resistance to ICIs reflect the diversity of tumor immune ecosystems, which can range from inflamed to immune-desert and immune-excluded patterns. Biomarkers such as PD-L1 expression, tumor mutational burden (TMB), and microsatellite instability (MSI) provide guidance but often fail to capture the full complexity of immune responsiveness, particularly across different histologies. Safety remains an equally important dimension: immune-related adverse events (irAEs) can range from mild cutaneous reactions to severe, occasionally irreversible autoimmune injury of vital organs [2,10]. Against this backdrop, circulating tumor DNA (ctDNA) and other liquid biopsy tools are emerging as dynamic biomarkers of minimal residual disease and treatment response, enabling earlier recognition of resistance and more timely adaptation of therapy [24]. In parallel, transcriptomic platforms that integrate tumor microenvironmental and immune signatures have begun to stratify responses to checkpoint blockade, for instance, in metastatic renal cell carcinoma, illustrating how multigene expression profiles can refine patient selection beyond single-analyte biomarkers [25].

Taken together, these developments point toward a more individualized use of immunotherapy, guided by biomarkers and ongoing assessment of response

Within this Special Issue, the featured articles mirror and extend these themes, covering topics that range from molecular targets and signaling pathways to cellular therapies and TME. Several contributions converge on checkpoint modulation and its crosstalk with oncogenic signaling. Morchón-Araujo et al. review inhibitory receptors such as LAG-3, TIM-3, and TIGIT, together with co-stimulatory molecules including OX40 and 4-1BB [26]. Their overview further encompasses engineered cytokines (IL-2 and IL-15), microenvironmental targets such as CCR8, the CD47–SIRPα axis, and TGF-β, as well as bispecific antibodies that simultaneously engage tumor antigens and T-cell receptors, underlining how checkpoint-based immunotherapy is diversifying into multilayered interventions across immune compartments [26].

In a complementary perspective, Cartwright et al. dissect the immunologic consequences of oncogenic signaling, showing that KRAS activation promotes the recruitment of MDSCs and induces CD47 expression, whereas BRAF mutations enhance PD-L1 levels. FGFR signaling can impair antigen presentation, while PI3K activation contributes to VEGF-driven angiogenesis and an immunosuppressive vasculature. These insights suggest that targeted therapies and ICIs should be carefully sequenced or combined, for example, by favoring immunotherapy-first strategies in BRAF-mutant melanoma to improve long-term outcomes [27].

The Special Issue also addresses immune targeting in particularly challenging clinical settings, including rare malignancies and engineered cell-based therapies. Galati et al. present an overview of blastic plasmacytoid dendritic cell neoplasm (BPDCN), a rare hematologic cancer marked by high expression of CD123 and CD303 [28]. Tagraxofusp, an IL-3–diphtheria toxin fusion protein, validated CD123 as a therapeutic target, though resistance frequently emerges. New strategies, antibody drug conjugates such as Pivekimab Sunirine, bispecific antibodies like Flotetuzumab, and CAR-T cells directed at CD123 or CD303, are under active development. Intriguingly, CD303, which has long been regarded as purely diagnostic, is being re-examined as a potential therapeutic target, particularly in combination with hypomethylating agents and BCL-2 inhibitors to potentiate responses [28].

In parallel, Stilpeanu et al. discuss how oncolytic viruses (OVs) may prime solid tumors for cellular therapies, promoting immunogenic cell death, increasing antigen release, inflaming the tumor milieu, and fostering T-cell infiltration and persistence—thereby potentially converting “cold” lesions into CAR-T-responsive tumors [29]. Complementing this view, Morimoto et al. report that systemic infusion of NK cells in glioblastoma xenograft models not only prolongs survival but also remodels the extracellular matrix reducing fibronectin and collagen VI while increasing the amount of chemokines such as CXCL14 and CCL19, suggesting a dual impact that couples direct cytotoxicity with stromal reprogramming [30]. Together, these findings underscore how viral platforms and NK-based strategies can be integrated with CAR-T approaches to breach microenvironmental resistance.

The primacy of tissue context, careful target selection, and robust biomarkers emerges with equal clarity in the remaining contributions. Moeller et al. show that neutrophil extracellular traps (NETs) in cutaneous squamous cell carcinoma (cSCC) exclude CD8^+^ T cells and associate with ulceration, strengthening the concept of immune-excluded tumors in which structural elements dictate immune access [31]. Patel et al. report the loss of SMARCB1 in lung squamous carcinoma among never-smokers, driving EZH2-mediated epigenetic silencing and conferring resistance to ICIs; in this setting, EZH2 inhibition may represent a rational strategy to restore sensitivity [32]. Likewise, Andryszak et al. identify prostate-specific membrane antigen PSMA expression in triple-negative breast cancer (TNBC), highlighting a potential biomarker and target in a subtype that has been historically labeled as antigen-scarce [33]. This microenvironment-centric perspective resonates with broader observations that tumor-associated macrophages (TAMs) are not immutable; rather, they can be pharmacologically reprogrammed toward pro-inflammatory, antigen-presenting states that synergize with existing immunotherapies and recondition the TME [34].

Downstream of target identification, Kashyap and Salman evaluate IL-13 receptor alpha 2 (IL-13Rα2) and ephrin type-A receptor 2 (EphA2) as targets in aggressive breast cancer, emphasizing both the opportunities and the challenges posed by antigen heterogeneity [35]. Finally, Galli et al. demonstrate that digital droplet PCR (ddPCR) enables highly sensitive quantification of CAR-T expansion, facilitating earlier insight into efficacy and toxicity and underscoring the growing importance of standardized molecular monitoring frameworks in cellular therapy [36].

These conceptual threads weave seamlessly into broader currents across immuno-oncology. Personalized, neoantigen-based mRNA vaccines, exemplified by Rojas et al.’s work on pancreatic cancer, suggest that tailored vaccination can reshape immune surveillance and synergize with checkpoint blockade and systemic therapies [23]. Noninvasive monitoring of ctDNA dynamics, as described by Nabet et al., enables earlier identification of benefit or resistance to immunotherapy and thus supports agile treatment adaptation [24]. Transcriptome-based signatures that integrate tumor-intrinsic, stromal, and immune features, as reported by Brown et al., can stratify responses to PD-1 blockade in metastatic renal cell carcinoma, anticipating a more widespread clinical role for multigene profiling [25]. Extending beyond single diseases, Sharifi et al. present a pan-cancer map of clinical cell-surface targets that are amenable to antibodies, antibody–drug conjugates, and cellular therapies, while warning that site-specific heterogeneity must guide precision trial design and therapeutic deployment [37]. Meanwhile, radiotherapy is being reimagined as an immunologic partner rather than a purely local modality: Lynch and colleagues highlight how its capacity to induce immunogenic cell death and enhance antigen release can be rationally combined with ICIs to drive systemic immune responses beyond the irradiated field [38]. Finally, Prelaj et al. synthesize an expanding toolkit of biomarkers, from ctDNA to radiomics and multi-omics, into artificial intelligence (AI)-enabled frameworks that could soon inform patient selection, treatment sequencing, and real-time adaptation in routine practice [39]

The overall message is not only that we now have more tools available, but that real progress depends on integrating immune effectors, genomic drivers, metabolic constraints, and stromal architecture within a single therapeutic framework. When viewed in this way, the near future of immuno-oncology will depend on converting biological and technological diversity into genuine therapeutic synergy. Durable benefit is likely to arise when immune checkpoints and engineered effectors are aligned with targeted inhibition of oncogenic pathways, metabolic reprogramming, and deliberate remodeling of TME, with each element coordinated and adjusted using high-resolution biomarkers and real-time monitoring tools [39]. Such approaches promise not only to refine patient selection and treatment sequencing but also to anticipate and counteract emergent resistance, enabling earlier, more rational adaptations. This Special Issue reflects this inflection point. By bringing together checkpoint biology and co-stimulatory signaling, oncogenic circuits and their immunologic consequences, cellular engineering and viral adjuvancy, and the analytics of liquid biopsy, transcriptomics, and AI, it offers a panoramic view of a field in motion: one that is converging on adaptive, personalized immunotherapy as its organizing principle. If these objectives are realized, immunotherapy may evolve from a powerful option into the central paradigm of precision oncology—reshaping not only how we treat cancer, but how we understand and measure response in individual patients in real time.

## Data Availability

Not applicable.
